# Portable bioluminescent platform for in vivo monitoring of biological processes in non-transgenic animals

**DOI:** 10.1038/s41467-021-22892-9

**Published:** 2021-05-11

**Authors:** Aleksey Yevtodiyenko, Arkadiy Bazhin, Pavlo Khodakivskyi, Aurelien Godinat, Ghyslain Budin, Tamara Maric, Giorgio Pietramaggiori, Sandra S. Scherer, Marina Kunchulia, George Eppeldauer, Sergey V. Polyakov, Kevin P. Francis, Jeffrey N. Bryan, Elena A. Goun

**Affiliations:** 1 Institute of Chemical Sciences and Engineering (ISIC), Swiss Federal Institute of Technology (EPFL), Lausanne, Switzerland; 2 Department of Chemistry, University of Missouri-Columbia, Columbia, MO USA; 3 Plastic and Reconstructive Surgery, Global Plastic Surgery, Lausanne, Switzerland; 4 Department of Neurosciences, University of Padova, Padova, Italy; 5 Institute of Cognitive Neurosciences, Free University of Tbilisi, Tbilisi, Georgia; 6 National Institute of Standards and Technology (NIST), Gaithersburg, MD USA; 7 Physics Department, University of Maryland, College Park, MD USA; 8 Department of Orthopaedic Surgery, David Geffen School of Medicine at UCLA, Santa Monica, CA USA; 9 Department of Veterinary Medicine and Surgery, University of Missouri-Columbia, Columbia, MO USA

**Keywords:** Bioluminescence imaging, Toxicology

## Abstract

Bioluminescent imaging (BLI) is one of the most powerful and widely used preclinical imaging modalities. However, the current technology relies on the use of transgenic luciferase-expressing cells and animals and therefore can only be applied to a limited number of existing animal models of human disease. Here, we report the development of a “portable bioluminescent” (PBL) technology that overcomes most of the major limitations of traditional BLI. We demonstrate that the PBL method is capable of noninvasive measuring the activity of both extracellular (e.g., dipeptidyl peptidase 4) and intracellular (e.g., cytochrome P450) enzymes in vivo in non-luciferase-expressing mice. Moreover, we successfully utilize PBL technology in dogs and human cadaver, paving the way for the translation of functional BLI to the noninvasive quantification of biological processes in large animals. The PBL methodology can be easily adapted for the noninvasive monitoring of a plethora of diseases across multiple species.

## Abstract (web summary)

Bioluminescence imaging tends to rely on transgenic luciferase-expressing cells and animals. Here the authors report a portable bioluminescent system to non-invasively measure intra- and extracellular enzymes in vivo in non-transgenic animals which do not express luciferase.

## Introduction

Recent advances in imaging technologies have revolutionized the fields of biomedical research, especially with respect to clinical diagnostics and drug discovery. Among the many known preclinical techniques, bioluminescence imaging (BLI) remains one of the most widely used due to its unprecedented sensitivity and ease of use. Current applications of BLI cover a wide range of therapeutic areas, in particular cancer and infectious diseases, but also including neurodegenerative, cardiovascular and metabolic disorders, such as diabetes and obesity^1–9^.

**Fig. 1:**
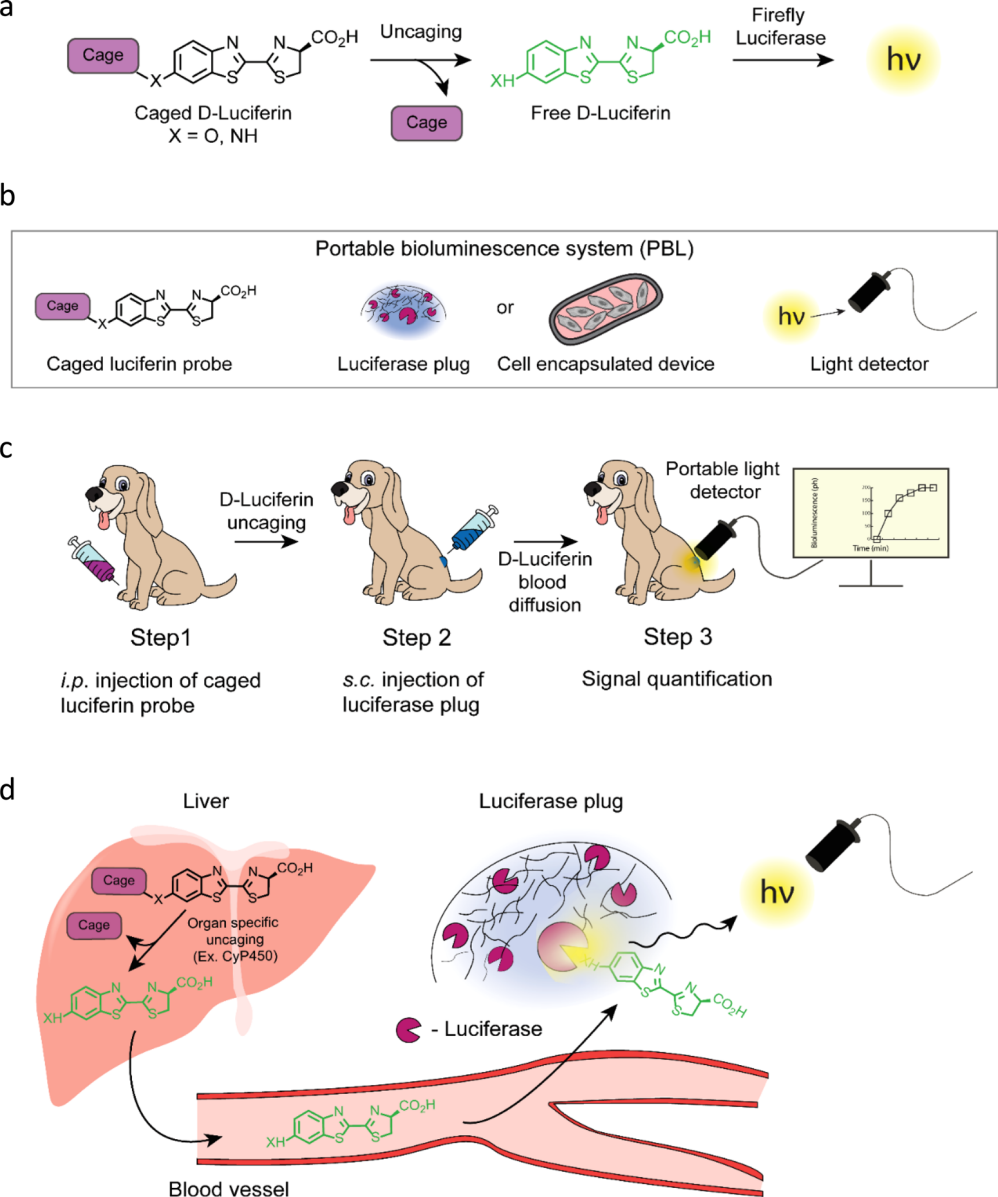
General concept of the PBL method for noninvasive in vivo monitoring of biological processes. **a** Basic principle of functional BLI. A bioluminescent probe with caged luciferin interacts with the targeted biological process. This reaction leads to uncaging of luciferin, which in turn is able to react with luciferase to produce bioluminescent light. Importantly, the amount of light generated as the result of this uncaging is proportional to the level of functional activity of the biological process of interest. **b** Three main components of the PBL system include (**a**) a caged luciferin probe that releases free luciferin by a specific biological process; **b** luciferase biodegradable plug or a cell encapsulating device with luciferase expressing cells that produces light proportionally to the amount of released luciferin; **c** sensitive portable light detector for signal quantification. **c** A typical PBL experiment employs an administration of a functional bioluminescent probe based on the caged luciferin scaffold followed by s.c. injection of a biodegradable luciferase plug. The light production is quantified by the portable light detector that is placed directly on top of the area of the plug. **d** Upon injection, a caged luciferin probe reaches the target organ (e.g., liver) where it gets uncaged by a specific biological process or enzyme (e.g., CyP450). Subsequently, free luciferin diffuses into the bloodstream and eventually reaches the luciferase containing plug. The luciferase enzyme in the plug produces light proportionally to the level of luciferin. The light is quantified by a sensitive light detector placed on top of the luciferase plug.

A typical BLI experiment employs a luciferase enzyme as a reporter that generates bioluminescent light upon oxidation of its substrate luciferin. Some of the first in vivo applications of BLI relied on the constitutive expression of luciferase enzyme in cancer cells to monitor tumor growth and metastasis^10,11^. More recently, caged luciferin-based probes were developed, opening-up the possibility of extending the application of BLI to the functional imaging of enzymatic and metabolic processes, including regulatory proteases; uptake of essential metabolites (e.g., glucose and fatty acids); fluxes of bioactive small molecules (e.g., H_2_O_2_ and H_2_S); cellular glycosylation; and intracellular delivery and release of therapeutically relevant compounds^3,6–9,12–26^. These functional BLI probes are based on the principle that chemically caged luciferin is not a substrate for luciferase until it becomes released or uncaged by a specific biological process of interest (e.g., selective enzymatic cleavage, Fig. 1a). The intensity of the bioluminescent signal quantitatively correlates with the amount of free luciferin in the animal, which in turn reflects the level of functional activity of a biological process of interest. Consequently, the acquired information reveals the dynamics of a wide range of biological functions that play key roles in physiological and pathological processes, as well as in drug discovery.

Despite all the advantages and widespread applications of BLI in the field of preclinical imaging, current BLI technology has a number of significant limitations. First, BLI is limited to use either in luciferase-expressing transgenic mice or in animals transplanted with luciferase-expressing cells^1–4,6–25^. While many relevant luciferase-expressing animal models have been developed in recent years, they still represent only a small percentage of existing in vivo models of human disease. Second, current in vivo imaging instruments usually comprise a small light-tight “black box” and a cooled charge-coupled device (CCD) camera as a light detector, making the technology relatively expensive, non-portable and, most importantly, restricting the use of BLI to small animals such as mice and rats. This latter size constraint represents a serious limitation to the use of BLI technology for drug development studies because many in vivo tests are performed on non-rodent animals such as dogs, cats, rabbits, and nonhuman primates. Third, the present-day instruments also require keeping animals under prolonged anesthesia during imaging, which can impact the animal’s health and be disruptive to its metabolism^27^. As a result of these limitations, many of the widely utilized animal experiments are still performed in a highly invasive way that leads to the sacrifice of a large number of animals. A striking example of such experiments is the toxicology testing of potential therapeutic candidates for the activation of cytochrome p450 (CYP450), a liver enzyme responsible for the deactivation of the majority of clinically used drugs. This one test alone leads to the sacrifice of hundreds of thousands of dogs each year^28^. Therefore, there is an urgent need for novel technologies that would allow noninvasive monitoring of biological processes in non-transgenic animals, especially large animals such as dogs.

To address the shortcomings of the current BLI technology and to expand its use to non-transgenic and non-rodent animals, we develop a “portable bioluminescent” (PBL) system. This system allows noninvasive measurements of biological processes in vivo using a luciferase-based biodegradable injectable “plug” in combination with a caged luciferin probe and a highly sensitive portable light detector. The PBL method can be easily applied for the imaging and quantification of a wide variety of biological processes for which caged luciferin probes already exist^3,4,6,8,9,18–25^. We choose to perform the validation studies using enzymatic processes as readout because there is a pressing demand for more efficient methods for in vivo evaluation of the activities of enzymes such as CYP450 in drug discovery studies. In addition to CYP450, which is an important example of an intracellular enzyme, we also investigate potential applications of this technology to therapeutically relevant extracellular enzymes. Dipeptidyl peptidase 4 (DPP-4) is selected as an example because of its essential role in the discovery of drugs for type 2 diabetes and several types of cancer^29–32^.

We first demonstrate that the PBL method is capable of noninvasive measurements of the activity of DPP-4 and CYP450 enzymes in vivo in non-luciferase-expressing mice. Importantly, the sensitivity and accuracy of the method are comparable with those obtained with standard stationary CCD optical imaging instruments (IVIS^®^ Spectrum). Next, we successfully apply PBL technology in dogs, paving the way for the translation of functional BLI to the noninvasive quantification of biological processes in large animals. Taken together, these results lay an important foundation for the potential replacement of highly invasive and destructive in vivo testing with the PBL methods that would help save millions of animal lives, especially large animals like dogs. Finally, we also demonstrate that PBL technology has the potential to be used in clinical settings by successfully quantifying bioluminescent signals directly in a human cadaver. This study demonstrates application of functional BLI technology for the noninvasive monitoring of biological processes in non-transgenic animals, laying an important foundation for replacing highly invasive tests with noninvasive readouts and for realizing the potential of such methods for clinical translation.

## Results

### General concept of the PBL method

The basic concept of the developed methodology is illustrated in Fig. 1 and Movie 1. The PBL system depicted on Fig. 1b consists of three main components: a functional bioluminescent probe, which is a caged luciferin compound that can sense a certain biological process of interest (e.g., CYP450 or DPP-4, Fig. 1a), a biocompatible luciferase-based bioluminescent light producing reporter “plug” or a cell encapsulating device, and a portable light detector. In a typical experiment, the animal is first injected with a single dose of caged luciferin probe followed by subcutaneous injection of the luciferase plug a few minutes later. Cell encapsulating device transplanted with luciferase-expressing cells is utilized for a long-term monitoring of biological processes (up to 5 months)^33^. The light detector is then immediately affixed on top of the luciferase plug and the bioluminescent signal is recorded at specific time intervals to obtain maximal light output (Fig. 1c). Upon injection of a caged probe, free luciferin is released in a target organ (e.g., liver) as a result of uncaging by a specific biological process or enzyme such as CYP450 (Fig. 1d). Free luciferin migrates into the bloodstream and eventually reaches luciferase-based reporter placed under the skin of test animal. The amount of light generated by the luciferase plug is proportional to the concentration of luciferin in the bloodstream, resulting in a bioluminescent light production that directly correlates with the level of functional activity of biological process of interest (e.g., enzymatic activity). In all of our experiments we utilized D-luciferin (Fig. 1a, X = O) that is referred as “luciferin” in the rest of the text. However, the same technology could be adapted for the use with other luciferases and their corresponding substrates such as Gaussia, NanoLuc and various red-shifted luciferases, which are all known to provide brighter in vivo signals^3,5,8,9,34–40^.

### Portable light detector

**Fig. 2:**
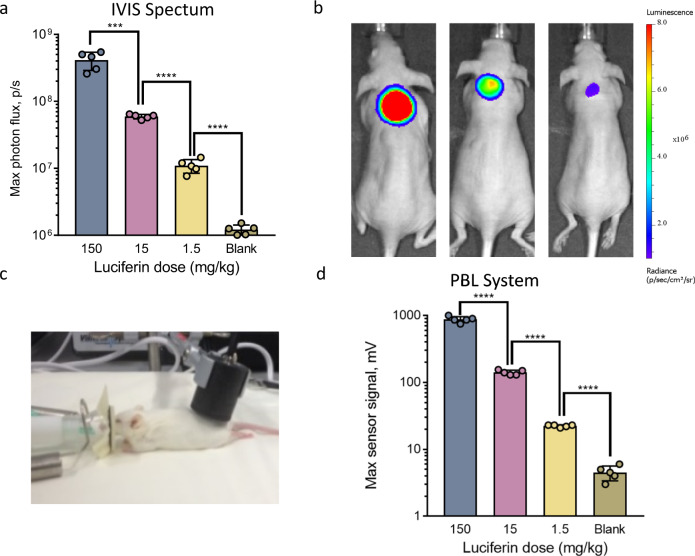
In vivo measurement of luciferin levels in non-transgenic animals using injectable luciferase plugs. Direct comparison of stationary CCD camera (IVIS^®^ Spectrum) and PBL readouts. **a** Maximal photon flux resulting from three groups of nude mice (*n* = 5) injected s.c. with 100 µL of luciferase plug in the dorsal area followed by i.p. injection of different doses of luciferin (1.5, 15, and 150 mg/kg) and imaging with the IVIS^®^ Spectrum. The “blank” group of mice was not injected with luciferin. **b** Representative images of mice from each luciferin-injected group described in (**a**). **c** Experimental setup for the PBL imaging experiment. The portable light detector is placed directly on the area of the luciferase plug upon administration of luciferin. **d** Maximum signal output from the experiment described in (**a**) except that the measurements were performed using the portable light detector. Data are presented as the mean ± s. d. (*n* = 5). Each “*n*” represents a biologically independent sample. Statistical significance (***P < 0.001; ****P < 0.0001) was calculated using a two-tailed unpaired *t*-test. Source data is available as a Source Data file for (**a** and **d**).

We developed a compact, portable large-aperture light detector with high sensitivity and low noise specifically for the detection of bioluminescent photon flux, which is typically relatively low. The design features a large 1 cm^2^ silicon photodiode in photovoltaic mode with a current-to-voltage transimpedance amplifier^41,42^ integrated in a cylindrical package with a diameter of 30 mm and height of 40 mm. Integrating the sensor with the amplifier helps to reduce the residual noise. The device resembles a stethoscope and can be easily applied to small and large animals as well as a human body (Fig. 2 and Fig. 5). The “working surface” of the device mechanically protects the sensitive electronic components and contains a circular optical aperture with 1 cm diameter. We used a commercial Hamamatsu photodiode S1227. The choice of a single-pixel photodiode over a camera is beneficial due to its low cost, room-temperature operation, and, most importantly, portability and low noise (low dark current). No spatial optical resolution is required because the detector is placed on top of a luciferase plug whose position is known. In addition, because the detector is placed directly on a light-emitting plug, most of the light emitted by the plug could be potentially detected. To detect low light levels, we used a low-noise operational amplifier configured as a current-to-voltage transimpedance amplifier, which is a commercial IC chip (Supplementary Fig. 1). The amplification factor was set to 10^10^ V/A with a feedback resistor *R* = 10 GigaOhm. The detector outputs a voltage reading that is proportional to optical radiant power absorbed by the diode surface: V = P/r, where P is radiant power, V is voltage and r is a proportionality coefficient called responsivity. To find r, we calibrate our detector. We also assess dark noise. Please refer to the *Methods* section for more detailed description of the detector technology.

### Luciferase-based injectable plug

The other component of the PBL method is the luciferase-based injectable plug (hereinafter referred to as a “luciferase plug”), which contains recombinant luciferase enzyme along with its cofactors and a polymeric matrix to keep the enzyme and its cofactors intact under the skin of the test animal (Fig. 1b, c, Movie 1). While the luciferase plug is designed for short-term monitoring of biological processes, previously reported cell encapsulating device should be utilized for the long-term studies (several months)^33^. To optimize the composition of the injectable luciferase plug and to achieve a bright stable signal in vivo, we tested the effect of different components on the light output using a stationary BLI instrument equipped with a sensitive CCD camera (IVIS^®^ Spectrum, Perkin Elmer). The data demonstrated that the luminescence generated by the luciferase plug is directly proportional to the amount of luciferase enzyme added to the plug and is relatively independent of the ATP concentration in the range of 1–10 mM (Supplementary Fig. 2a, b). As a result of this study, the following composition of the luciferase plug was chosen for all further experiments in mice: 83 μL of Matrigel^®^, 10 μg of luciferase enzyme, 10 mM ATP, 1 mM Mg^2+^ and PBS up to a 100 μL total volume. The Matrigel^®^ matrix was selected for this study because it is nontoxic, easy to implant by s.c. injection, and produced brighter and more stable signal compared to other matrices (Supplementary Fig. 2c). Matrigel^®^ based plug was stable for at least 60 min post s.c. injection enabling continuous measurements of bioluminescent signal and determination of maximal light output (Supplementary Fig. 3a). These data also demonstrate that repetitive measurements taken more than 1 h apart in the same animal will require reinjection of the new plug to achieve better consistency of the results.

Since the surface light intensity is dependent on the depth of the light source, we decided to quantify the dependency of the bioluminescent light output on the depth of the luciferase plug. We used the cuts of meat from a butcher to quantify signal loss to a measured tissue thickness (Supplementary Fig. 3b). As expected, the intensity of the detected light was dependent on the depth of the light source. Interestingly, the drop in the signal intensity was not as dramatic as we expected with the regular firefly luciferase (about tenfolds per 0.8 cm of meat). We could still see a clear signal even at the depth of 1 cm, suggesting that this method is suitable for the use with a wide range and concentrations of luminescence imaging probes^6,7,9,12–25^. Since the surface signal is dependent on the depth of the plug, the consistency of the plug injection across study groups is very important factor for achieving the best reproducibility of the results.

### Quantification of blood luciferin levels with luciferase plug

To investigate whether the amount of light generated by the luciferase plug is proportional to the concentration of luciferin in the blood of test animals, we injected different concentrations of luciferin solution intraperitoneally (i.p.) into nude mice followed by s.c. injection of luciferase plugs in the dorsal side of the test animals. The animals were then immediately anesthetized and placed into the stationary CCD camera (IVIS^®^ Spectrum). The bioluminescent light output was continuously measured to determine the maximal optical radiant power (measured in phtons/sec and referred to as “maximum photon flux”). Our data demonstrate that the maximal photon flux resulting from the luciferase plug linearly correlates with the amount of injected luciferin within a large dynamic range of three orders of magnitude (150, 15 and 1.5 mg/kg doses), suggesting that the plug can be utilized for accurately quantifying luciferin concentrations in the blood of test animals (Fig. 2a). The representative images of mice injected with the luciferase plug and three different concentrations of luciferin are shown on Fig. 2b.

The same set of experiments was then performed using the portable light detector, with the procedure for bioluminescence measurement similar to that described above using the IVIS^®^ Spectrum (the experimental setup is shown in Fig. 2c). The results demonstrated in Fig. 2d show a similar linear correlation between the luciferin concentration and the maximal optical power measured by the portable light detector. Moreover, the error bars shown in Fig. 2a and Fig. 2d are also comparable. These data indicate that the PBL method is suitable for the sensitive quantification of the free luciferin concentration in the blood of non-transgenic animals that do not express the luciferase enzyme. Importantly, the signal linearity correlates with the amount of injected luciferin over a large dynamic range (three orders of magnitude −3 logs).

### Measurements of enzymatic activities in living mice

**Fig. 3:**
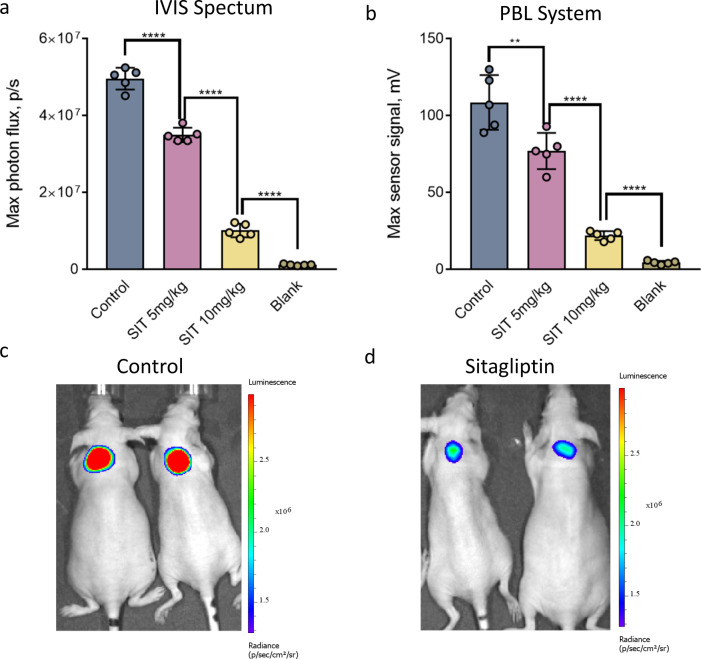
Noninvasive in vivo measurements of extracellular enzymatic activity (DPP-4) in non-transgenic mice: direct comparison of stationary CCD camera (IVIS^®^ Spectrum) and PBL readouts. **a** Maximal photon flux obtained from four groups of nude mice (*n* = 5) resulting from administration of a DPP-4-specific caged luciferin probe^12^ and DPP-4 inhibitor sitagliptin (“SIT”). The “SIT” groups of mice received an oral gavage of different concentrations of SIT (5 and 10 mg/kg) 30 min prior to injection of the probe, while the control group received a gavage of vehicle only (PBS buffer). All the mice received s.c. injection of 100 µL of luciferase plug in the dorsal area followed by signal acquisition with the IVIS^®^ Spectrum. The “blank” group of mice received injection of the plug without caged luciferin probe. **b** Maximum signal output from the experiment described in (**a**) except that the measurements were performed using the portable light detector. **c–d** Representative images of two mice from the control (**c**) and 10 mg/kg sitagliptin-treated (**d**) groups. Data are presented as the mean ± s. d. (*n* = 5). Each “n” represents a biologically independent sample. Statistical significance (****P < 0.0001) was calculated using a two-tailed unpaired *t*-test. Source data is available as a Source Data file for (**a** and **b**).

To evaluate whether this PBL method can be used for accurate measurements of biological processes using functional bioluminescent probes, we first performed a feasibility study with a caged luciferin probe previously designed by us for selective sensing of the activity of DPP-4^12^, a representative example of an extracellular enzyme. Four cohorts of Swiss nude mice (*n* = 5) were used for this study. Two different doses of 5 and 10 mg/kg of the selective DPP-4 inhibitor sitagliptin (abbreviated as “SIT”) were administered by oral gavage in PBS buffer to two group of animals, while the third group of mice was treated with PBS only (control). Thirty minutes post gavage, three groups of mice received an injection of the DPP-4 caged luciferin probe followed by s.c. injection of the luciferase plug 10 min post injection of the probe. The last group of mice was injected with the plug but not the caged luciferin probe and therefore was used as a negative control (blank). The signal was acquired from all four groups of mice using either the IVIS^®^ Spectrum or portable light detector. A strong bioluminescent signal was observed from the control group of mice treated with the DPP-4 caged luciferin probe alone with both the IVIS^®^ Spectrum and portable light detector (Fig. 3). Importantly, the dose dependent reduction in the signal obtained from the animals treated with the DPP-4 inhibitor was observed, and these measurements were fully consistent between the IVIS^®^ Spectrum and portable light detector readouts (Fig. 3a, b). These results suggest that the PBL methodology is able to provide an accurate readout of extracellular enzymatic activity in non-transgenic animals in a noninvasive fashion, and that the acquired data is comparable to that obtained with the established stationary “black box” CCD cameras technology.

**Fig. 4:**
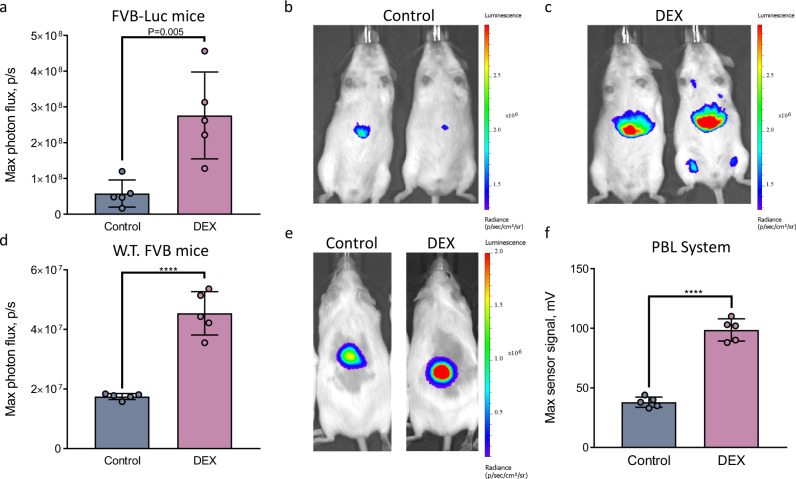
Noninvasive in vivo measurements of intracellular enzymatic activity of CYP450: direct comparison of stationary CCD camera (IVIS^®^ Spectrum) and PBL readouts. **a** Maximal photon flux resulting from two groups of FVB-luc^+/+^ mice (*n* = 5) injected with the Luciferin-IPA^TM^ probe, a caged luciferin reagent specifically designed to measure activation of the Cyp3a isozyme of CYP450^45^. The “DEX” group of mice was treated with dexamethasone, a known activator of Cyp3a, 24 h prior to the injection with the Luciferin-IPA^TM^ probe, while the control group received a gavage of vehicle only (vegetable oil). The light output was measured with the IVIS^®^ Spectrum. **b–c** Representative images of two mice from the control (**b**) and DEX-treated (**c**) groups. **d** Maximal photon flux obtained from two groups of wild-type non-transgenic FVB mice (*n* = 5) injected i.p. with the Luciferin-IPA^TM^ probe followed by injection of the luciferase plug (s.c. in the dorsal area) and subsequent imaging by the IVIS^®^ Spectrum. Analogous to the experiment described in (**a**), the “DEX” group of mice was treated with DEX 24 h prior to the injection with the Luciferin-IPA^TM^ probe while the control group received the gavage of vehicle only. The light output of the plug was measured with the IVIS^®^ Spectrum. **e** Representative mouse images from the control and DEX-treated groups in the experiment described in (**d**). **f** Maximum signal output from the experiment described in (**d**) except that the measurements were performed using the portable light detector. Data are presented as the mean ± s. d. (*n* = 5). Each “n” represents a biologically independent sample. Statistical significance (****P < 0.0001) was calculated using a two-tailed unpaired *t*-test. Source data is available as a Source Data file for (**a**, **d** and **f**).

Next, we examined the feasibility of PBL to measure the activity of an intracellular enzyme, such as CYP450, in vivo. We first investigated whether the activity of CYP450 can be detected in vivo under conditions previously reported to trigger activation of the enzyme, namely, upon treatment with a xenobiotic dexamethasone^43^. While many different isozymes of CYP450 are known, we decided to specifically focus on cytochrome P450 3A (abbreviated Cyp3a), which is the most common and versatile isozyme involved in drug metabolism^44^. The caged luciferin probe specific to this isozyme is fully validated and commercially available (Luciferin-IPA^TM^, Promega), along with many other cytochrome specific luciferins^45^. Two cohorts of age-matched genetically engineered mice that ubiquitously express luciferase through the beta-actin promoter (FVB-luc^+/+^ mice) were used for this study^46^. The experimental group of mice was injected with dexamethasone (abbreviated as “DEX”, 50 mg/kg dose i.p.), which was previously shown to specifically cause acute activation of Cyp3a^47,48^, while the control group of mice was treated with vehicle alone (vegetable oil). After 24 h, both groups received i.p. injections of the Luciferin-IPA^TM^ probe, followed by anesthesia of the animals and signal acquisition using the IVIS^®^ Spectrum. As shown in Fig. 4a–c, the bioluminescent signal from the DEX-treated mice was approximately three times higher than the signal from the control group, indicating that the probe can successfully detect Cyp3a activation directly in vivo upon treatment of mice with DEX.

Having confirmed the feasibility of measuring Cyp3a using IVIS technology, we repeated the above experiment using non-transgenic animals and our PBL methodology. Two groups of wild-type FVB mice were used, one of which was injected with DEX and the other of which was injected with vehicle (control). After 24 h, the Luciferin-IPA^TM^ probe was injected i.p. into both groups of mice. The mice were then anesthetized, injected with a luciferase plug and imaged with either the IVIS^®^ Spectrum or portable light detector. As shown in Fig. 4d–f, a significantly higher bioluminescent signal was observed in the animals treated with DEX than in the vehicle-treated control mice using both the IVIS^®^ Spectrum (Fig. 4d-e) and portable light detector (Fig. 4f).

In order to compare the imaging results with the conventional measurements of cytochrome activity, we isolated liver samples from wild type and DEX treated FVB-Luc mice and performed qPCR analysis of Cyp3a expression. The results of qPCR analysis showed similar folds increase in Cyp3a expression (Supplementary Fig. 3c). These data suggest that the current invasive and time-consuming methods of measuring Cyp3a activity can be successfully replaced by the noninvasive bioluminescent detection methodologies, especially PBL, which, as shown below, is fully translatable to large animal studies.

### Application of the PBL method for noninvasive bioluminescent signal quantification in large animals and a human post-mortem model

Since the main goal of the PBL method was the translation of functional BLI to large animals, we next decided to investigate whether this methodology would work in a canine model. While luciferin had been extensively used at relatively high concentrations in mice (up to 750 mg/kg)^49^ with no obvious signs of adverse effects^49–54^, no such data were reported for large animals like dogs. Thus, we performed a serum chemistry blood toxicology analysis upon administration of a clinically relevant amount of luciferin in healthy dog (i.p. injection of 15 mg/kg). We specifically measured the levels of alanine aminotransferase (ALT), alkaline phosphatase (ALP), gamma-glutamyltransferase (GGT), serum creatine kinase (CK), glutamate dehydrogenase (GLDH) and total bilirubin in a commercial laboratory before and 24 h after administration of luciferin. These serum biomarkers are routinely utilized to detect drug-induced liver injury in humans and experimental animals^55,56^. While increased ALT serum levels generally correlate with hepatocytes damage, induction of serum ALP reflects the extent of injury to the biliary epithelial cells. Also, increase in total bilirubin is indicative of hepatic functional impairment or processing of bilirubin production (hemolysis)^55^. We also measured the levels of GLDH that is a mitochondrial matrix enzyme responsible for amino acid oxidation and urea production^57^. In addition, recent clinical studies demonstrated high diagnostic potential of this GLDH test in predicting hepatic toxicity in patients with various liver pathologies and therefore it has been proposed as more sensitive and specific biomarker of liver injury than ALT^56^. Lastly, we investigated changes in GGT and CK levels that all have been previously used as a diagnostic markers of drug-induced liver injury^58–60^. Our data shown in Supplementary Table 1 clearly demonstrate that no clinically relevant elevation was observed in any of these parameters, indicating lack of D-luciferin toxicity in the dog at the concentration studied.

**Fig. 5:**
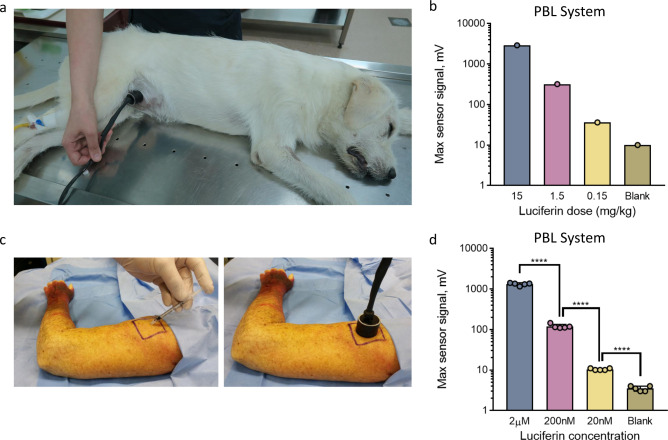
Noninvasive BL signal quantification in large animals (dogs) and a human post-mortem model using the PBL method. **a** Experimental setup for the application of the PBL method in dogs. Three large dogs were anesthetized followed by s.c. injection of 0.5 mL of the luciferase plug in the abdominal area followed by i.p. injection of 5 mL of luciferin solution at three different concentrations (15 mg/kg, 1.5 mg/kg and 0.15 mg/kg). The portable light detector is then placed on the area of the luciferase plug, and measurements are obtained for 30 min following the luciferin injection. The experiment was performed as a proof of principle with *n* = 1 for each time point with one luciferin injection per dog. **b** Maximal signal output obtained from dogs injected with different concentrations of luciferin (15, 1.5 and 0.15 mg/kg in 5 mL of PBS). **c** Experimental setup for PBL measurements in a human post-mortem model. The luciferase plug matrix was premixed with three different doses of luciferin (2 µM, 200 nM and 20 nM) followed by direct s.c. injection of the 100 µL of the activated luciferase plug under the skin of a human cadaver. The portable light detector was assembled directly on top of the plug followed by signal acquisition for 15 min. Three independent injections were performed for each luciferin concentration. **d** Average maximal signal output obtained by the portable light detector in the experiment described in (**c**). Statistical significance (**** *P* < 0.0001) was calculated using a two-tailed unpaired *t*-test. Source data is available as a Source Data file for (**b** and **d**).

Inspired by these results, we investigated whether the amount of light generated by the luciferase plug is proportional to the concentration of injected luciferin in dogs. The dogs were anesthetized, and the luciferase plug was implanted subcutaneously in the ventral abdomen. Various concentrations of luciferin (15, 1.5 and 0.15 mg/kg) were then administered via i.p. injection followed by the placement of the portable light detector directly on the area of the luciferase plug (Fig. 5a). As shown in Fig. 5b, the maximal light output linearly correlated with the amount of injected luciferin within a large dynamic range of three orders of magnitude. Due to strict regulation on animal experimentation in large animals, this experiment was performed as a proof of principle with *n* = 1 per time point. These results suggest that the PBL method can be utilized for accurate measurements of the luciferin concentrations in the blood of large animals such as dogs, and that the acquired data were fully consistent with the previous data obtained in mice (Fig. 2). Under the same experimental conditions, the maximal sensor signal in dogs was ~30 times higher than that in the mouse experiments (15 mg/kg dose; Fig. 2). These data lay an important foundation for the replacement of highly invasive biological tests in large non-rodent animals with noninvasive measurements using the PBL method.

Finally, we investigated whether PBL technology has the potential to be translated to human diagnostic applications. For these experiments, we premixed 100 µL of luciferase plug with three different doses of luciferin (2 µM, 200 nM and 20 nM final concentrations) followed by direct s.c. injection of the mixture under the skin of a human cadaver in the area of the upper arm (Fig. 5c). The portable light detector was then placed directly on top of the luciferase plug, and the signal was acquired for 15 min. A significant signal was observed even at the lowest luciferin concentration, which provided a signal threefold higher than the background signal (no plug). Moreover, the signal was proportional to the luciferin concentrations in the range of three orders of magnitude (Fig. 5d). These data demonstrate that the PBL technology has full potential to work successfully in humans, paving the way for the translation of BLI all the way to the clinic.

## Discussion

In comparison to conventional BLI, the PBL method provides a major technological breakthrough for a number of reasons. It allows the analysis of a wide range of biological processes by completely removing the need to use luciferase engineered cells or transgenic animals. This feature dramatically expands the scope of BLI to many existing animal models of human diseases and opens-up the opportunity for the use of this powerful modality in large animals and potentially humans. Importantly, this work lays the foundation for the replacement of current highly invasive large animals tests performed by utilizing noninvasive readouts, which falls in-line with the requirements of the 3 R principles of animal experimentation. For example, CYP450 is a major detoxicant of xenobiotics, and measurements of its activity are routinely performed by multiple drug development companies as a part of toxicological studies. The procedure involves the administration of high doses of xenobiotics followed by multiple blood withdrawals, after which the animals must be sacrificed. The blood is then analyzed by HPLC-MS methods that usually provide only a few data points per animal with rather large error bars due to multiple sample manipulation steps^61^. In contrast, bioluminescent readouts have been shown to provide much higher sensitivity and better kinetic parameters than those obtained from end-point assays^62,63^, However, to date such bioluminescent readouts have not been adapted for activity measurements of CYP450 in live animals despite the fact that a full range of caged luciferin probes for a variety of different CYP450 isozymes have been previously reported^45^. Our data in mice clearly demonstrate that bioluminescence readouts can be used for accurate noninvasive quantification of the activity of specific isozymes of CYP450 with both PBL technology and standard CCD cameras and have full potential to be translated to large animals such as dogs.

In the current study, we utilized a minimally invasive “injectable” approach for the development of a luciferase-based biodegradable plug for measuring the luciferin concentration in the blood of living animals. This approach is ideally suited for the measurements of relatively fast biological processes such as the activation of certain enzymes. However, PBL can also be extended to long-term longitudinal measurements of biological processes by repetitive injections of the luciferase plug, since it is composed of nontoxic and biodegradable material. Moreover, luciferin has been used extensively in preclinical settings with repeated injections of rather high doses (up to 750 mg/kg) with no mention of any harmful side effects^49^. In addition, it was reported that the growth rate of luciferase-labeled cell lines in syngenic mouse models monitored by repetitive injections of high doses of luciferin is identical to the growth rates of wild type cells, suggesting that luciferin is not toxic to animals and the immune response to luciferase is extremely weak, if immunogenic at all^64,65^. We also performed an extensive blood toxicology analysis before and after luciferin injection in dog and did not observe elevation of any clinically relevant parameters indicating lack of luciferin toxicity at concentration studied.

Alternatively, the luciferase plug can be replaced with a cell-encapsulated device that remains a stable source of luciferase for up to 5 months^33^. Indeed, such fully s.c. implanted devices have already been developed and widely used in humans for passive immunization against Alzheimer’s disease by providing stable delivery of recombinant anti-amyloid-β antibodies^66^ and for intrathecal delivery of ciliary neurotrophic factor in amyotrophic lateral sclerosis patients^67^. These cell encapsulating devices are small in size and require only minimal surgical interventions^33,66,67^. In addition, novel technologies in the field of electrochemical and optical sensors have allowed the creation of s.c. implantable devices that can wirelessly send data to mobile devices and have already been used in clinical practice for the continuous monitoring of glucose levels in humans^68^. These recent technological advances allow future translation of the PBL method to portable and fully implantable “to be worn” biosensors for in vivo longitudinal measurements of a wide range of biological processes.

While we utilized a predominantly naturally derived firefly luciferin-luciferase system in the current study, we also envision the use of the PBL method with other recently developed substrates such as CycLuc1^34^, AkaLumine^5,38^, naphthyl-luciferins^36^, and several others^3,5^ that provide much brighter in vivo signals with firefly luciferase due to increased tissue penetration of red-shifted light emission. The method can also be optimized to be used with other luciferases such as red-shifted click beetle luciferase, Gaussia luciferease and NanoLuc^3,5,8,9,34–40^. Both of the latter luciferases should be very effective for use with the PBL method as they are extremely bright and can significantly increase sensitivity of the assay. Indeed, secreted Gaussia luciferase was previously used to monitor gene expression through a simple blood sampling in non-transgenic animals to monitor angiogenesis^69^. In addition, many caged coelenterazines substrates have been reported in the literature^37^.

The portable nature of the PBL method also offers additional advantages over conventional techniques, such as avoiding the need to constrain the animals in the light-tight “black box” (e.g., IVIS^®^ Spectrum) and the use of anesthesia. Both of these factors are often associated with additional stress to the animal and have been reported to interfere with biological readouts, such as brain function, cardiac activity, and general metabolism^70–72^. Another important advantage of PBL technology is its extremely low cost (~500–600 USD), which is orders of magnitude lower than that of existing stationary “black box” CDD cameras, making the PBL method an ideal choice for studies that do not require spatial resolution. For example, the anatomical location of many enzymes is organ or tissue specific, such as liver specific CYP450, various cancer proteases, or gut microbiota enzymes. In addition, the location of a biological event can be further controlled by the route of administration of a functional bioluminescent probe (e.g., oral gavage for studies on the gastrointestinal absorption of metabolites or functions of gut microbiota)^18,20,73^.

In conclusion, the PBL method overcomes all the major limitations of BLI and provides a major advancement of the powerful BLI modality toward many fundamental and therapeutic applications. Most importantly, it allows the quantification of multiple biological processes directly in non-transgenic animals in a noninvasive and simple-to-use fashion. Moreover, our data demonstrate that PBL technology should be translatable to virtually any large animal for the sensitive quantification of biological processes using functional bioluminescent readouts. This ability also opens-up the potential for using PBL in veterinary settings for routine diagnostic testing of companion animals (e.g., noninvasive monitoring for treatment of liver or kidney failure in dogs and cats). The portable and cost-effective features of PBL technology, combined with its exquisite sensitivity and quantifiability, make this powerful tool a strong candidate for adoption in numerous areas of preclinical and clinical research and diagnostics.

## Methods

### Reagents

Matrigel^®^ was purchased from Corning, USA (cat. # 356237); ATP disodium salt was purchased from AppliChem GmbH, Germany (cat. # A1348-0005); MgSO_4_ ·7H_2_O – AlfaAesar, Germany (cat. # A14491-0B); PBS – ThermoFisher Scientific, USA (cat.# 10010-015); recombinant firefly luciferase – Sigma-Aldrich (cat.# SRE0045); D-luciferin – Perkin Elmer, USA (cat.# 122799); DEX – Sigma-Aldrich (cat.# 31375); QIAGEN RNeasy kit - QIAGEN, Switzerland; Q-Gel - QGel SA, Switzerland; and collagen, DEX, sitagliptin – Sigma-Aldrich. DPP-4-specific caged luciferin probe (DAL) and Luciferin-IPA^TM^ were chemically synthesized according to the published protocols^12,45^.

### Portable light detector

For light detection we used a large-area (1 cm^2^) silicon photodiode, which is a single-pixel device. The photodiode generates electrical current in response to irradiation, and the electrical current is proportional to the optical power that is absorbed by the diode surface. The current is converted to voltage with a low-noise transimpedance amplifier. The output voltage is read by a stock voltmeter connected to the detector by a BNC cable, see Supplementary Fig. 1 for more details. Our detector design is inspired by the accurate light detection method used for standard transfers^41,42^. To measure responsivity, we calibrate our portable detector with a visible light source at 632 nm using a substitution calibration method. Our portable detector is compared to the trap detector with a known detection efficiency^74^. The measured responsivity is 3.32(3) × 10^9^ V/W. To measure dark noise, we block the detectors optical input and obtain a series of voltage readings with an integration time of 2 s (as it is done in the experiment). We measure the dark noise of 6 × 10^−14^ W RMS. Therefore, we can resolve an input optical flux of ~200,000 photons of visible light per second with signal-to-noise ratio of one. In certain cases, it might be appropriate to integrate for 1 min or longer, further reducing detection noise to about 1.1 × 10^−14^ W RMS, which would yield resolving a flux of ~35,000 photons of visible light per second with signal-to-noise ratio of one. To convert optical power expressed in watts to that expressed as photon flux, we use Planks formula: F = Pλ_0_/hc, where F is photon flux, P is power, h is Planks constant, c is the speed of light, and λ_0_ is the weighted average wavelength of the bioluminescence emission spectrum. Unlike an imaging device, this detector is placed directly on the animal at the injection site of the luciferase plug, so that it collects nearly all of the bioluminescent light emitted from ~1 cm^2^ skin surface.

### Luciferase-based injectable plug

The following optimized composition of the luciferase plug was used for the experiments: 83 μL of Matrigel^®^ matrix, 10 μg of luciferase enzyme (1 × 10^11^ units per mg, Sigma-Aldrich SRE0045), 10 mM ATP, 1 mM Mg^2+^ and PBS up to a 100 μL total volume. ATP, Mg^2+^, luciferase and PBS were first premixed in an Eppendorf tube, followed by the addition of ice-cold Matrigel^®^ matrix. The concentrations of stock solution were as follows: ATP – 100 mM, Mg^2+^ −0.5 M and luciferase - 10 μg/μL. Typically, 1.2 mL of plug solution was prepared and stored at −20 °C until use and 100 μL of the plug solution was used for each mouse injection and experiments in human cadaver. Subcutaneous injection was performed using 1 mL syringe. The size of the plug post injection was ~7 × 7 mm. 500 μL of the plug solution was used for experiments in dogs.

### Optimization of luciferase-based injectable plug

The optimal plug composition was developed by testing different concentrations of key components of the plug such as ATP, matrix, and luciferase enzyme. In order to optimize ATP concentration, plugs containing 10 μg of luciferase enzyme, 1 mM Mg^2+^ and Matrigel^®^ were supplemented with 1, 5 or 10 mM of ATP. In order to optimize the amount of luciferase enzyme, plugs containing 10 mM ATP, 1 mM Mg^2+^ and Matrigel^®^ were supplemented with 10 or 100 µg of pure luciferase enzyme. Different matrices including Matrigel^®^, q-GEL and Collagen 1 were tested in plugs containing 10 μg of luciferase enzyme, 1 mM Mg^2+^ and 10 mM ATP. Nude mice were injected with 150 mg/kg dose of luciferin in PBS followed by s.c. injection of different plug formulations at the dorsal side of the mouse. The signals were obtained using IVIS^®^ Spectrum instrument.

### Experimental animals

We purchased FVB-luc^+/+^ mice (full abbreviation: FVB-Tg[CAG-luc, GFP]L2G85Chco/J) from Jackson Laboratory and Swiss nu/nu mice from Charles River Labs. All animal BLI experiments were reviewed and approved through a license VD2994, VD2849c from the Swiss Cantonal Veterinary Office Committee for Animal Experimentation according to the Swiss National Institutional Guidelines. All in vivo imaging mouse experiments were performed in at least five animals, and the results were quantified either using Perkin Elmer Living Image^®^ software (for IVIS^®^ Spectrum images) or a stock voltmeter. The standard deviation was calculated using Excel STDEV function. *P* values were calculated as a two-tailed *t*-test using GraphPad Prism v7.03 software.

### Measurements of blood luciferin levels in non-transgenic mice using injectable luciferase plugs

Different doses of luciferin (150 mg/kg, 15 mg/kg or 1.5 mg/kg in 100 μL of PBS) were administered i.p. to the test animals. Ten minutes post injection of luciferin, 100 μL of the liquid form of the luciferase plug was injected subcutaneously at the dorsal side of the nude mice. The mice were then anesthetized, placed into an IVIS^®^ Spectrum imaging instrument and imaged with automatic settings. A series of sequential images was acquired over a period of 15 min following the plug injection. Alternatively, the animals were placed in a dark box, and the sensor was positioned on top of the luciferase plug. Each experiment was performed with at least five animals per group.

### Noninvasive in vivo measurements of DPP-4 activity

Four cohorts (*n* = 5) of Swiss nude mice were used for this study. The experimental groups was treated with 5 and 10 mg/kg dose of sitagliptin in 100 µL of PBS by oral gavage. The control group was treated with 100 µL of PBS. After 30 min, these groups of mice were injected with a DPP-4 probe as previously described^12^. Briefly, mice were injected i.v. with 200 μL of 30 mM GPc peptide in PBS (55 mg/kg). In 15 min the mice injected i.p. with 100 μL of 10 mM (5.9 mg/kg) CBT in 30% v/v PEG400:70% water. After another 10 min, the animals were anesthetized, injected s.c. with 100 µL luciferase plug and immediately imaged using either the IVIS^®^ Spectrum (*n* = 5) or portable light detector (*n* = 5) over a period of 15 min. The last group of mice was injected with the plug but not caged luciferin probe (blank) and imaged immediately after injection of the plug.

### Noninvasive in vivo measurements of CYP450 activity

Two cohorts of FVB-luc^+/+^ mice (*n* = 5) were used for the first experiment to establish the possibility of direct imaging of Cyp3a activation in vivo using the Luciferin-IPA^TM^ probe. The experiment layout was based on a previously published procedure^45^. The experimental cohort of mice was injected i.p. with DEX (50 mg/kg dose in 100 µL of vegetable oil), and the control cohort was injected with vegetable oil alone (vehicle). After 24 h, all mice were injected with 0.5 mg of the Luciferin IPA^TM^ probe, anesthetized and imaged with the IVIS^®^ Spectrum over a period of 15 min. In the next experiment, we used two groups of wild-type FVB mice (*n* = 5). The experiment was conducted using exactly the same procedure as that for the FVB-luc^+/+^ animals, except that the 100 μL of luciferase plug matrix was s.c. injected into the dorsal side of the mice 10 min following the injection of the Luciferin-IPA^TM^ probe followed by imaging with the IVIS^®^ Spectrum over a period of 15 min. Next, the same experiment was repeated on the other two groups of wild-type FVB mice (*n* = 5) except that the portable light detector was used to acquire the bioluminescent signal.

### qPCR analysis of Cyp450 expression

Liver tissues were collected from wild type and DEX treated mice, mRNA was isolated using QIAGEN RNeasy kit, and gene expression was measured by real-time PCR. Two micrograms of RNA were used to make first strand of cDNA, followed by real-time qPCR using SYBR® Green Master Mix (Applied Biosystems) in an Applied Biosystems QuantStudio 3 sequence detector system. The results of cycle threshold were plotted into the standard curve separately using Applied Biosystems QuantStudio 3 software and the final value of the target gene was normalized to mouse 18 s. The list of qPCR primers is provided in Supplementary table 2.

### Noninvasive bioluminescent signal quantification in dogs using the PBL method

The study was approved by the Bioethics Committee of the Ivane Beritashvili Center of Experimental Biomedicine (#13/08122017). Larger plugs and lower luciferin concentrations were used in this study than in the mouse study. Three dogs were anesthetized and 0.5 mL of luciferase plug solution was injected s.c. into the hairless abdominal region of the dog as depicted on Fig. 5a followed by i.p. injection of different doses of luciferin in 5 mL of sterile PBS with one luciferin injection per dog (15 mg/kg, 1.5 mg/kg and 0.15 mg/kg). The bioluminescent signal was then acquired over a period of 30 min using the portable light detector. The animals were kept in a dark room without any additional insulation of the light sensor.

### Noninvasive bioluminescent signal quantification in a human post-mortem model using the PBL method

The study was approved by the Direction of Human Morphology Core facility of the Faculty of Biology and Medicine of the University of Lausanne (UNIL-CHUV), Switzerland. The body was donated to the University of Lausanne, Switzerland for medical research purposes with the consent of the donor. One hundred microliters of luciferase plug solution was mixed with different luciferin concentrations in PBS (2 µM, 200 nM and 20 nM final concentrations) and injected subcutaneously into the test subject in the area of the upper arm. The portable light detector was then placed directly on top of the luciferase plug, and the resulting signal was acquired over a period of 15 min.

### In vivo stability of luciferase based Matrigel^®^ plug

Standard plugs were injected s.c. in the dorsal region of nude mice. The mice were injected with 150 mg/kg dose of luciferin in PBS at different time points after injection of the plug (5 min, 30 min, 1 h, 2 h, and 5 h), and immediately imaged with IVIS Spectrum over a period of 15 min.

### Dependency of surface light intensity on the depth of the signal source

The dependency of light detection on the depth of the source signal was investigated using the standard luciferase plug. The plug was directly mixed with 2 µL of 1 µM luciferin solution, immediately added to a well of a 96 well plate and imaged using IVIS Spectrum (baseline signal). Several slices of about 2 mm retail ham were then consequently placed over the well, and the plate was imaged after addition of each slice.

### Blood toxicology analysis

Serum samples from a healthy dog before and 24 h post 15 mg/kg i.p. injection of 5 mL of luciferin solution in sterile PBS were analyzed using standard analytical techniques. The following parameters were measured: alanine ALT, ALP, GGT, serum CK, GLDH and total bilirubin concentrations.

### Reporting summary

Further information on research design is available in the Nature Research Reporting Summary linked to this article.

## Supplementary information

Supplementary Information

Description of Additional Supplementary Files

Supplementary Movie 1

Reporting Summary

## Data Availability

The data that support the findings of this study are available from the corresponding author upon reasonable request. Source data are provided with this paper.

## Source data

Source Data
