# Supplementary material for: Portable bioluminescent platform for in vivo monitoring of biological processes in non-transgenic animals
Source: Nat Commun. 2021 May 11;12:2680. doi: 10.1038/s41467-021-22892-9 (PMC8113525; doi:10.1038/s41467-021-22892-9)
Supplement: Supplementary file 1 — Supplementary Information [file 41467_2021_22892_MOESM1_ESM.pdf]

Supplementary Figures

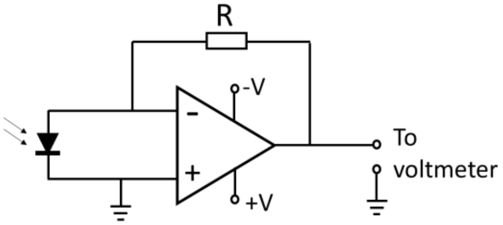

Supplementary Figure 1. Scheme of the portable light detector. The photodiode is connected to a trans-impedance amplifier comprised of a low-noise operational amplifier and a feedback resistor R (here R=10^{10} Ohm). The output voltage is measured using a conventional voltmeter. The operational amplifier uses two DC voltage sources (+V=15 Volt and -V=-15 Volt).

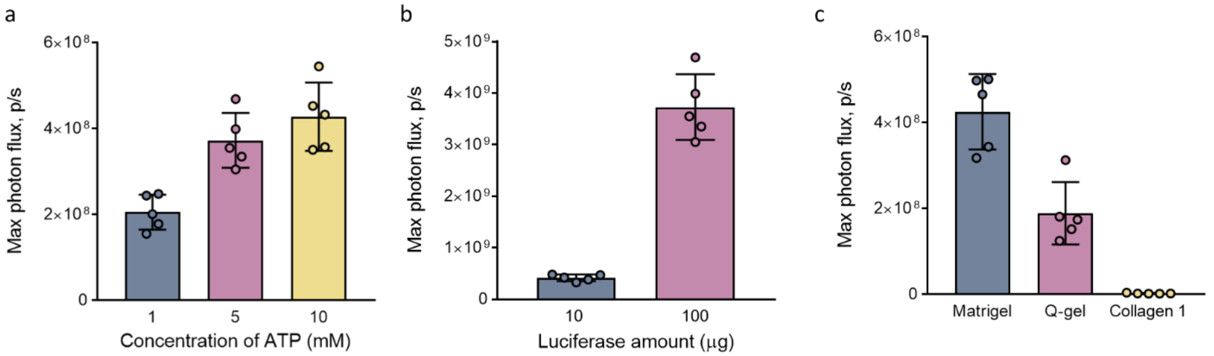

Supplementary Figure 2. Optimization of the composition of biodegradable luciferase plug. a) Plugs containing 10 μg of luciferase enzyme, 1 mM Mg^{2+} and Matrigel® were supplemented with different concentrations of ATP (1, 5, and 10 mM). Nude mice were injected with 150mg/kg dose of luciferin followed by s.c. injection of the plugs at the dorsal side of the mouse. The signal was obtained using IVIS® Spectrum instrument. Data are presented as the mean +/- s. d. (n=5). Each "n" represents a biologically independent sample. **b)** Plugs containing 10 mM ATP and 1 mM Mg^{2+} were supplemented with different amounts of luciferase enzyme (10 μg or 100 μg). Nude mice were injected with 150 mg/kg dose of luciferin followed by s.c. injection of the plugs

at the dorsal side of the mouse. The signal was immediately acquired using IVIS® Spectrum. **c)** Plugs containing 10 μg of luciferase enzyme, 1 mM Mg^{2+} and 10 mM ATP mixed with different biodegradable matrices. Nude mice were injected with 150 mg/kg dose of luciferin followed by s.c. injection of the plugs at the dorsal side of the mouse. The signal was immediately acquired using IVIS® Spectrum. Data are presented as the mean +/- s. d. (n=5). Each “n” represents a biologically independent sample.

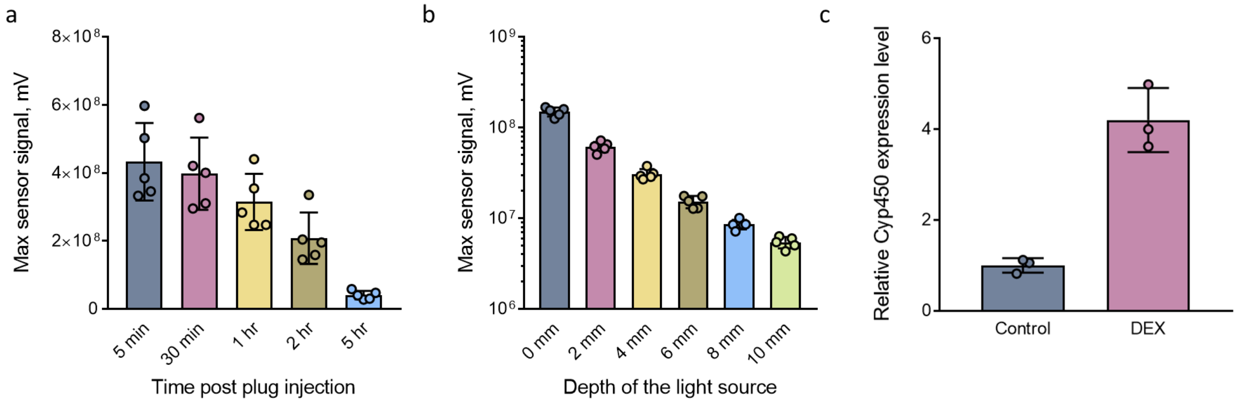

Supplementary Figure 3. a) Stability of the Matrigel® based luciferase plug. Standard plugs were injected s.c. at the dorsal side of the mouse, followed by the i.p. injection of 150 mg/kg dose of luciferin at various time points post injection of the plug. The signal was obtained using IVIS® Spectrum instrument. Data are presented as the mean +/- s. d. (n=5). Each “n” represents a biologically independent sample. **b)** Dependency of the detected light intensity on the depth of the light source. Standard luciferase plugs were mixed with luciferin solution and added to a well of a 96 well plate. The plate was covered by increasing numbers of ~2 mm retail ham slices and imaged with IVIS® Spectrum instrument. Data are presented as the mean +/- s. d. (n=5). Each “n” represents a biologically independent sample. **c)** qPCR expression analysis of CyP450 expression in normal and DEX treated liver samples. The expression of the cytochrome was normalized to the expression of mouse ubiquitin. Data are presented as the mean +/- s. d. (n=3).

Supplementary Tables

Assay	Before Luciferin	After Luciferin	Reference Interval
Total Bilirubin	0.2 mg/dL	0.1 mg/dL	0.1 - 0.4
ALT	22 U/L	23 U/L	14 - 76
ALP	61 U/L	61 U/L	12-98
GGT	4 U/L	3 U/L	0-8
CK	83 U/L	85 U/L	40 - 226
GLDH	5 U/L	5 U/L	no ref.

Supplementary Table 1. Blood toxicology analysis before and after administration of D-luciferin to a dog at 15 mg/kg.

Primer Name	Sequence
mouse Cyp3a11 F	GACAAACAAGCAGGGATGGAC
mouse Cyp3a11 R	CCAAGCTGATTGCTAGGAGCA
mouse 18s F	AGTCCCTGCCCTTTGTACACA
mouse 18s R	GATCCGAGGGCCTCACTAAAC

Supplementary Table 2. List of qPCR primers
