# Supplementary material for: Portable bioluminescent platform for in vivo monitoring of biological processes in non-transgenic animals
Source: Nat Commun. 2021 May 11;12:2680. doi: 10.1038/s41467-021-22892-9 (PMC8113525; doi:10.1038/s41467-021-22892-9)
Supplement: Supplementary file 2 — Description of Additional Supplementary Files [file 41467_2021_22892_MOESM2_ESM.pdf]

Title: Supplementary Movie 1

Description: The scientific Movie 1 demonstrates how the PBL technology works and outlines some important biological applications.
