# Supplementary material for: Portable bioluminescent platform for in vivo monitoring of biological processes in non-transgenic animals
Source: Nat Commun. 2021 May 11;12:2680. doi: 10.1038/s41467-021-22892-9 (PMC8113525; doi:10.1038/s41467-021-22892-9)
Supplement: Supplementary file 4 — Reporting Summary [file 41467_2021_22892_MOESM4_ESM.pdf]

nature research

Corresponding author(s): Elena Goun

Last updated by author(s): Mar 17, 2021

Reporting Summary

Nature Research wishes to improve the reproducibility of the work that we publish. This form provides structure for consistency and transparency in reporting. For further information on Nature Research policies, see our Editorial Policies and the Editorial Policy Checklist.

Statistics

For all statistical analyses, confirm that the following items are present in the figure legend, table legend, main text, or Methods section.

n/a	Confirmed	
☐	☒	The exact sample size (n) for each experimental group/condition, given as a discrete number and unit of measurement
☐	☒	A statement on whether measurements were taken from distinct samples or whether the same sample was measured repeatedly
☐	☒	The statistical test(s) used AND whether they are one- or two-sided<br>*Only common tests should be described solely by name; describe more complex techniques in the Methods section.*
☒	☐	A description of all covariates tested
☒	☐	A description of any assumptions or corrections, such as tests of normality and adjustment for multiple comparisons
☐	☒	A full description of the statistical parameters including central tendency (e.g. means) or other basic estimates (e.g. regression coefficient) AND variation (e.g. standard deviation) or associated estimates of uncertainty (e.g. confidence intervals)
☐	☒	For null hypothesis testing, the test statistic (e.g. F, t, r) with confidence intervals, effect sizes, degrees of freedom and P value noted<br>*Give P values as exact values whenever suitable.*
☒	☐	For Bayesian analysis, information on the choice of priors and Markov chain Monte Carlo settings
☒	☐	For hierarchical and complex designs, identification of the appropriate level for tests and full reporting of outcomes
☒	☐	Estimates of effect sizes (e.g. Cohen's d, Pearson's r), indicating how they were calculated

Our web collection on statistics for biologists contains articles on many of the points above.

Software and code

Policy information about availability of computer code

Data collection	Living Image 4.1 (Perkin Elmer), Applied Biosystems QuantStudio 3
Data analysis	Living Image 4.1, Microsoft Word 2016, Microsoft Excel 2016, Applied Biosystems QuantStudio 3, GraphPad Prism 7.03

For manuscripts utilizing custom algorithms or software that are central to the research but not yet described in published literature, software must be made available to editors and reviewers. We strongly encourage code deposition in a community repository (e.g. GitHub). See the Nature Research guidelines for submitting code & software for further information.

Data

Policy information about availability of data

All manuscripts must include a data availability statement. This statement should provide the following information, where applicable:
- Accession codes, unique identifiers, or web links for publicly available datasets
- A list of figures that have associated raw data
- A description of any restrictions on data availability

The data that support the findings of this study are available from the corresponding author upon request

Field-specific reporting

Life sciences study design

All studies must disclose on these points even when the disclosure is negative.

Sample size	No sample-size calculations were performed. Sample size was determined to be adequate based on the magnitude and consistency of measurable differences between groups.
Data exclusions	No data were excluded
Replication	All experiments were repeated 2-3 times (except for the proof of principle experiment in dogs where n=1). All attempts at replication were successful.
Randomization	Mice and dogs were allocated to the study groups randomly. No cell culture experiments were performed in this study.
Blinding	Blinding would not affect the results and was not relevant to the study. All the measurements were performed using instruments.

Reporting for specific materials, systems and methods

We require information from authors about some types of materials, experimental systems and methods used in many studies. Here, indicate whether each material, system or method listed is relevant to your study. If you are not sure if a list item applies to your research, read the appropriate section before selecting a response.

Materials & experimental systems

n/a	Involved in the study	
☒	☐	Antibodies
☒	☐	Eukaryotic cell lines
☒	☐	Palaeontology and archaeology
☐	☒	Animals and other organisms
☐	☒	Human research participants
☒	☐	Clinical data
☒	☐	Dual use research of concern

Methods

n/a	Involved in the study	
☒	☐	ChIP-seq
☒	☐	Flow cytometry
☒	☐	MRI-based neuroimaging

Animals and other organisms

Policy information about studies involving animals; ARRIVE guidelines recommended for reporting animal research

Laboratory animals	Female nude mice, 6-8 weeks old, female FVB-Tg[CAG-luc,-GFP]L2G85Chco/J mice, 6-12 weeks old, female wt FVB mice, 6-8 weeks old. Dogs: 3 males ages 3, 7 and 8. Mice were kept under 12 hr dark/light cycle, 23 C temperature and 40-60% humidity.
Wild animals	No wild animal involved
Field-collected samples	No field samples collected
Ethics oversight	All animal experiments performed with mice were approved by the Veterinary Authority of the Canton Vaud, Switzerland (License VD2849c, VD2994); The study in dogs was approved by the Bioethics Committee of the Ivane Beritashvili Center of Experimental Biomedicine, Tbilisi, Georgia (#13/08122017)

Note that full information on the approval of the study protocol must also be provided in the manuscript.

Human research participants

Policy information about studies involving human research participants

Population characteristics	N/A
Recruitment	The body was donated to the University of Lausanne, Switzerland for medical research purposes with the consent of the donor.
Ethics oversight	The study was approved by the University of Lausanne, Switzerland

Note that full information on the approval of the study protocol must also be provided in the manuscript.
